# Intralobar pulmonary sequestration with bronchial atresia and a systemic artery feeding a normal contralateral lung

**DOI:** 10.1259/bjrcr.20150176

**Published:** 2015-11-12

**Authors:** Marica Giannotta, Maria Barbara Leone, Laura Greco, Michelangelo Baldazzi, Maurizio Zompatori

**Affiliations:** Department of Specialized, Diagnostic and Experimental Medicine, Sant'Orsola-Malpighi Hospital, University of Bologna, Bologna, Italy

## Abstract

We report a case of an intralobar pulmonary sequestration in the left lower lobe presenting with aspects of bronchial atresia associated with an anomalous systemic artery supplying the basal segments of the right lower lobe, which showed no bronchial or parenchymal anomalies. This is an extremely rare occurrence and has been reported only twice in the literature so far.

## Summary

Pulmonary sequestration is an uncommon congenital anomaly caused by disrupted embryogenesis resulting in a non-functioning area of the lung that is not in communication with the bronchial tree whose arterial feeding comes from the systemic circulation, mostly through the aorta.^1^ Pulmonary sequestration is divided into intralobar and extralobar. Intralobar pulmonary sequestration represents 75% of all sequestrations usually affecting the left lower lobe; the arterial supply is commonly via the descending aorta.^2^ We report a very rare case of intralobar pulmonary sequestration in the left lower lobe associated with a systemic arterial supply from descending thoracic aorta to the basal segments of the right lower lobe, presenting a normal bronchial tree.

## Case report

**Figure 1.**
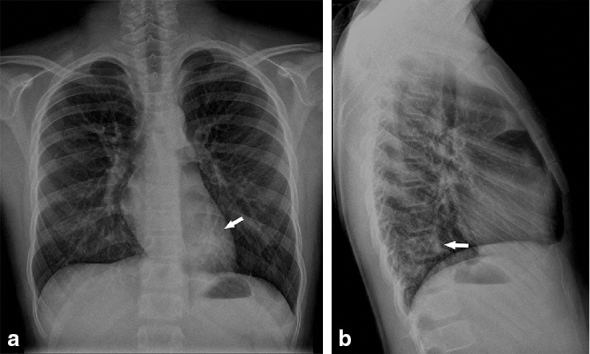
Chest radiograph, (a) postero-anterior and (b) latero-lateral views. A lung consolidation (arrows) is visible in the left retrocardiac region.

**Figure 2.**
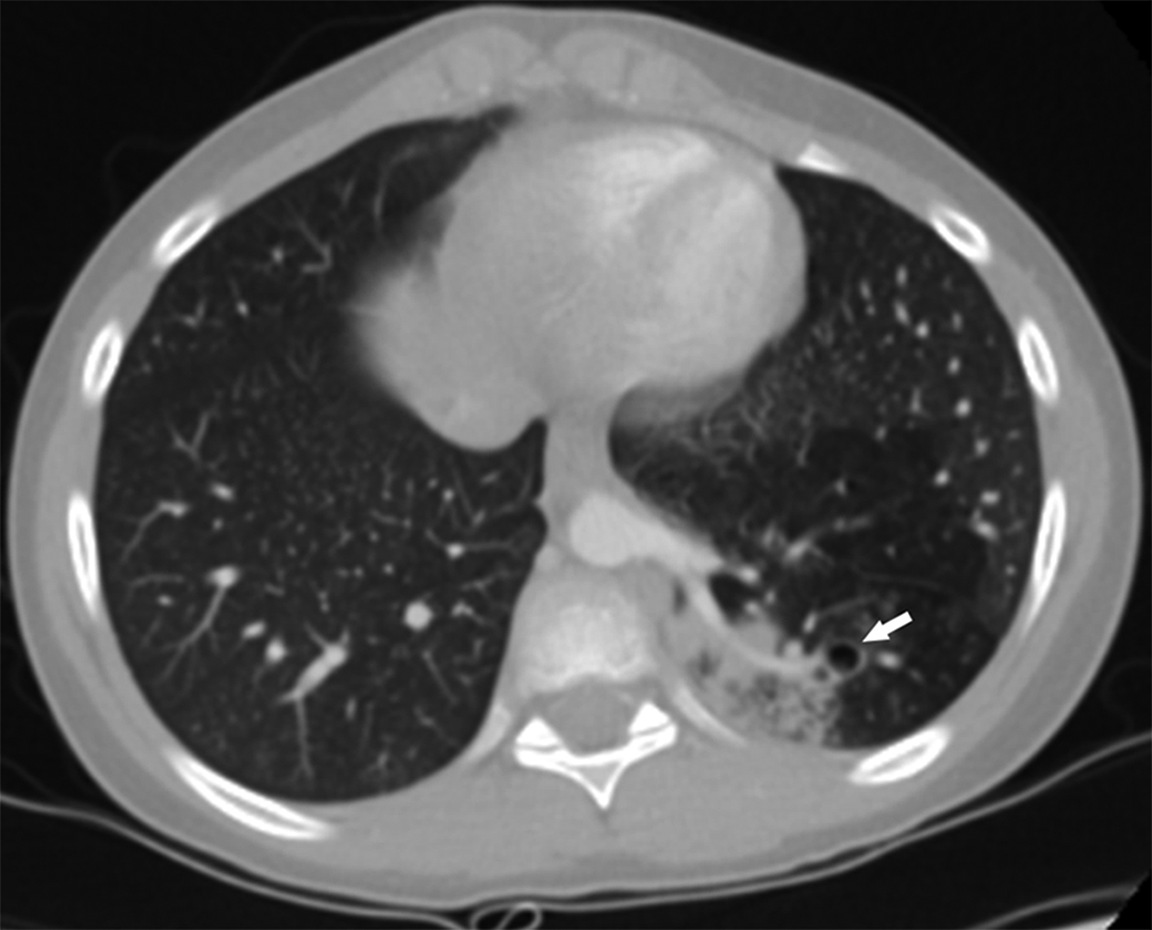
Axial CT scan through the lower lobe of the left lung in the lung window. An anomalous artery reaches a dysplastic parenchymal area in the left lower lobe posterior segment, corresponding to intralobar pulmonary sequestration (arrow); consolidation and trapping are concurrent.

**Figure 3.**
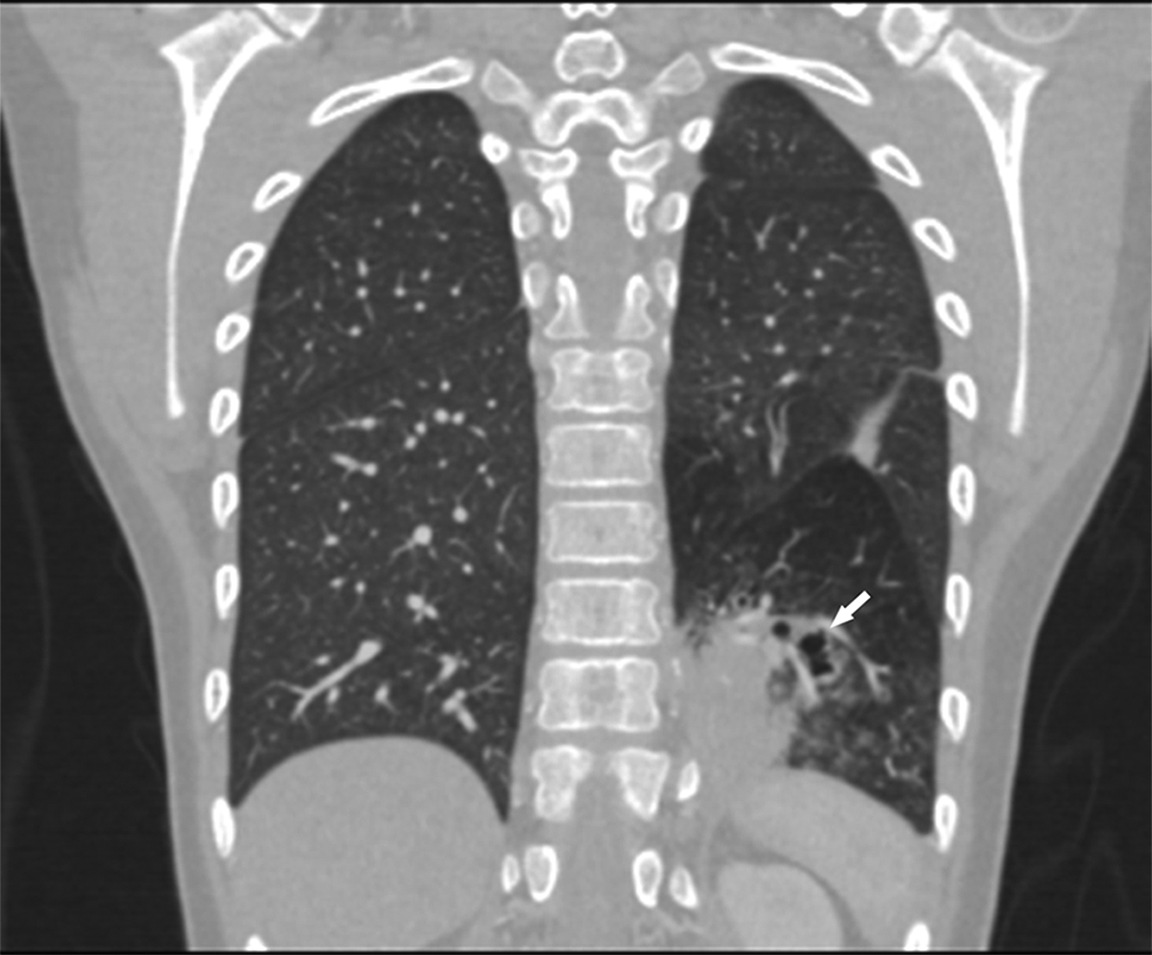
Coronal thoracic CT scan better showing the heterogeneous consolidation not communicating with the respiratory tract in the left lower lobe posterior segment (arrow).

**Figure 4.**
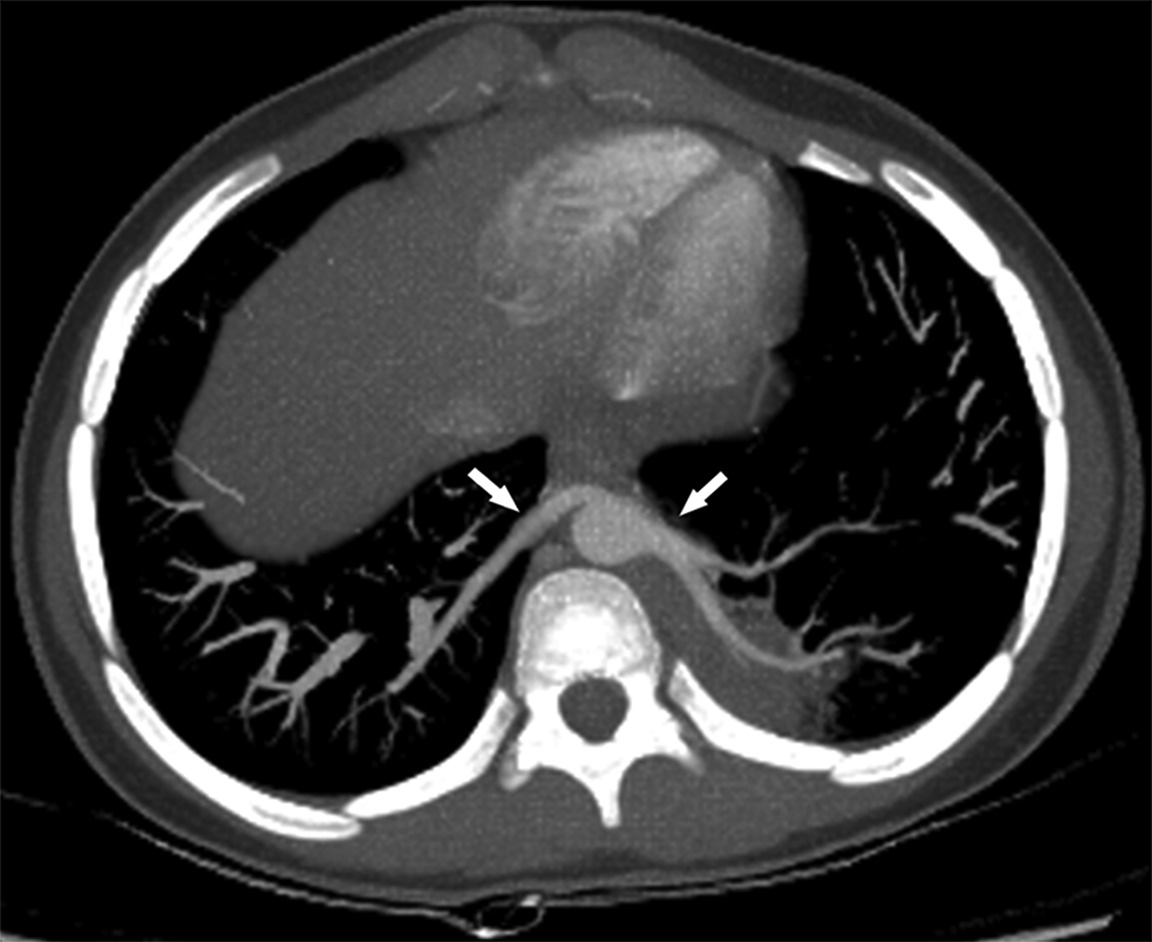
Contrast-enhanced study shows two anomalous vessels originating from the distal portion of the descending thoracic aorta: one vessel going from the posterior lateral portion of the aorta to the left lower lobe and another originating in the anterior area and extending to the right lower lobe (arrows).

**Figure 5.**
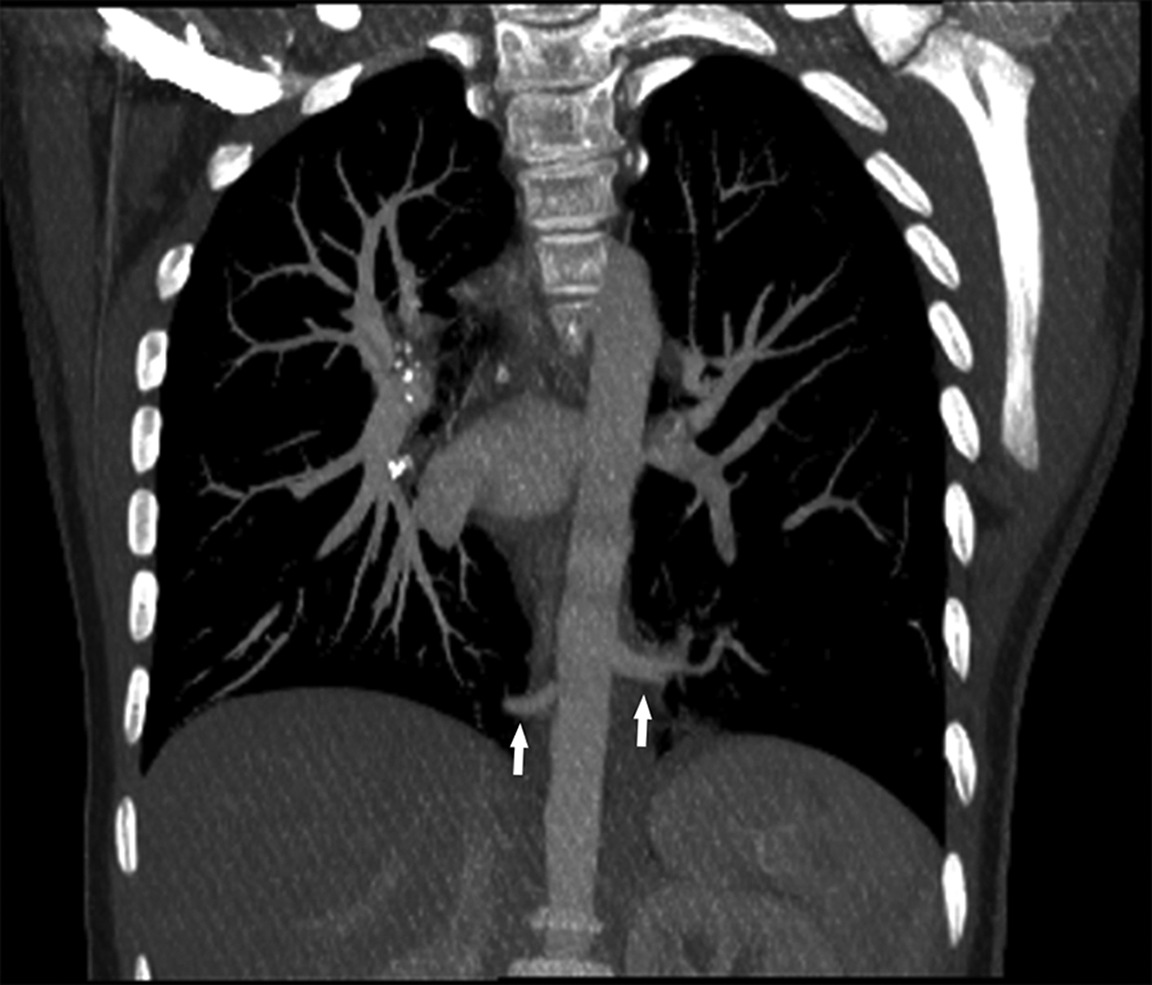
Coronal maximum intensity projection reconstruction, showing the origin of the two anomalous arteries from the descending aorta (arrows).

**Figure 6.**
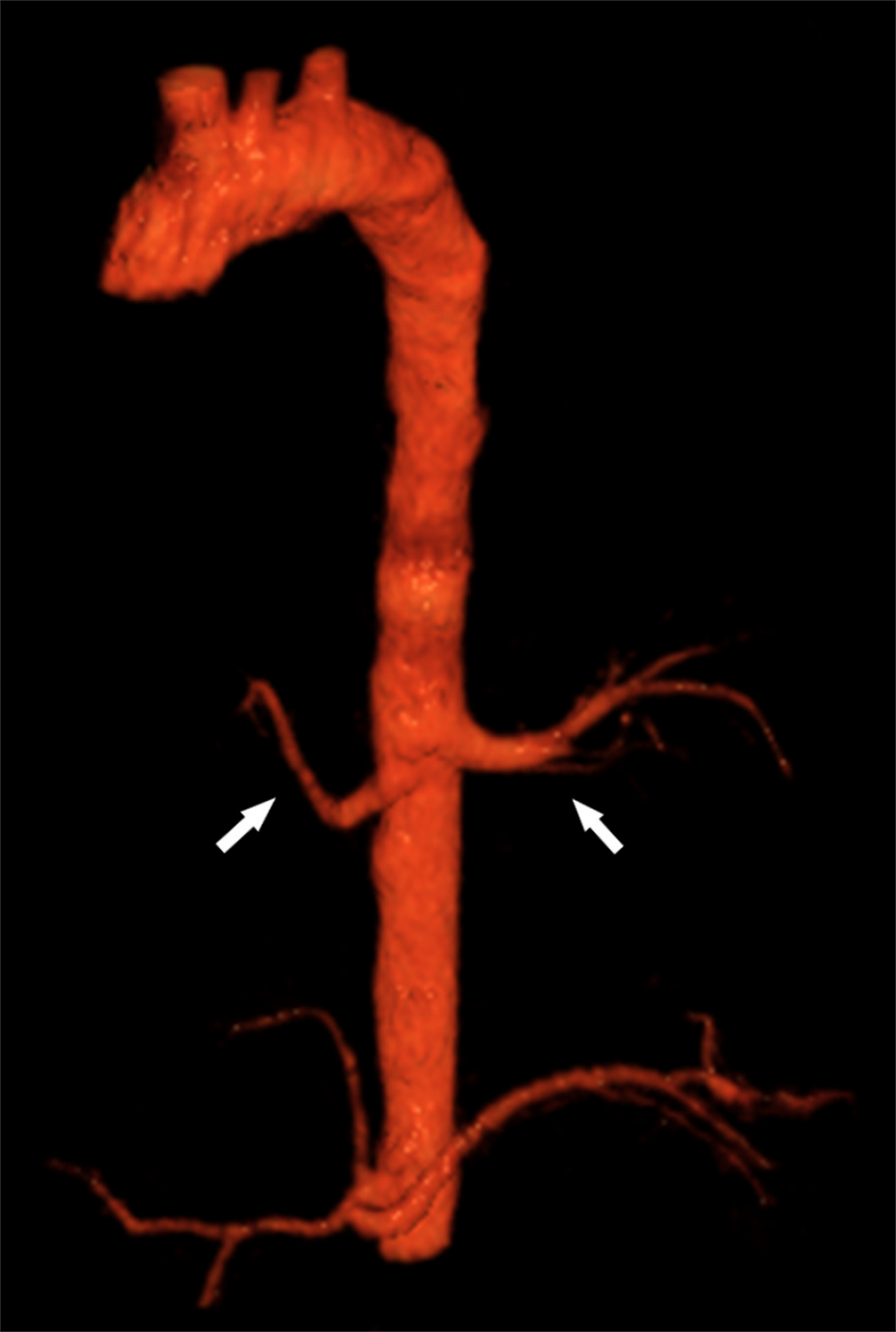
Volume rendering reconstruction of thoracic aorta better showing the emergence of the two anomalous arteries (arrows).

In February 2014, an 11-year-old male presented with a history of recurrent respiratory tract infections and suspected tuberculosis following a case within his family. On physical examination, he appeared to be in good health. Chest auscultation did not reveal any specific pathological heart or lung sounds. Abdominal objectivity was negative. His parents did not refer any other relevant medical history about him. Tuberculin skin tests resulted positive and a chest X-ray showed a dishomogeneous parenchymal consolidation in the left lower lobe (Figure 1a,b). Suspecting active tuberculosis, a preliminary CT scan of the thorax was performed showing a heterogeneous consolidation with some cystic masses containing mixed fluid and air in the left lower lobe posterior segment, not in communication with the respiratory tract, and a probable expression of a dysplastic parenchymal area (Figures 2 and 3). These findings were suggestive of pulmonary sequestration, including a differential diagnosis of congenital pulmonary airway malformation, because of the presence of a cystic component within the consolidation. The injection of contrast medium showed an artery arising from the descending thoracic aorta that divided into two 1.2 cm after its exit, with both branches extending to the dysplastic area; therefore, a diagnosis of intralobar sequestration with associated aspects of bronchial atresia was made; superinfection and trapping of contiguous parenchyma coexisted. Just below the emergence of the previously anomalous vessel, another artery was detected that crossed the midline to achieve a healthy parenchyma in the right pulmonary base, configuring a pattern of aberrant systemic artery feeding a normal lung (Figures 4–6). Other congenital anomalies that appear to be related to pulmonary sequestration^3^ were absent; in particular, pulmonary venous drainage was regular through the pulmonary veins, there was no communication between the bronchus and the oesophagus, and no diaphragmatic defects or other gross pulmonary anomalies were identified either. Our patient responded well to antitubercular antibiotic therapy and his clinicians, together with his family, decided to keep him under control with clinical follow-up, avoiding surgery for the moment.

## Discussion

Pulmonary sequestration was classified by Pryce^4^ as intra- and extra-lobar based on their morphological patterns; intralobar form is a dysplastic area of lung tissue in the context of normal parenchyma sharing a common visceral pleura. Extralobar sequestration is coated by its own pleura. However, as a large number of variants of pulmonary sequestration has been described, not all adapt to the original definition. Sade et al^5^ introduced the term pulmonary sequestration spectrum in order to propose a group of congenital anomalies that seem to be related and share a common embryogenesis. Therefore, this spectrum includes various combinations of bronchial, vascular and other anomalies that may or may not be present, such as bronchial sequestration of the pulmonary parenchyma, arterial supply from the systemic circulation, anomalous pulmonary venous drainage towards the right atrium, communication between the bronchus and the oesophagus, defects of the diaphragm and gross pulmonary anomalies such as horseshoe lung or hypoplasia.^3^ Systemic vascularization of the normal lung has been classified as “Pryce Type I sequestration”^4^ in which there are no bronchial or parenchymal anomalies and the lung is perfused by pulmonary and systemic arteries. This last anomaly mostly affects the left lower lobe and less commonly the right lower lobe.^3,6,7^ Intralobar sequestration is more frequent than extralobar (75% *vs* 25%); both usually affect the left side, intralobar in 60% of cases with predominant involvement of posterior basal segment and extralobar in 90% of cases;^1^ commonly, their systemic blood supply is from the aorta but less frequently, it can be from intercostal, subclavian, internal thoracic or pericardial arteries.^2^ Usually the diagnosis of intralobar pulmonary sequestration is incidental during imaging examinations performed for other reasons: the patients frequently present a history of recurrent bronchitis or pneumonia, more rarely of haemoptysis and only in 15% of cases, it can be asymptomatic. Extralobar pulmonary sequestration, on the other hand, is mainly found in neonates and less frequently in late infancy or early childhood. Patients become symptomatic because of respiratory infection, displacement of the organs or when a complication occurs.^1^ Anomalous systemic arterial supply to normal lung is clinically asymptomatic in most cases. Haemoptysis is the most frequent clinical feature. Other signs and symptoms can include exertional dyspnoea, lower thoracic murmur and congestive heart failure owing to left heart overload.^8^ Regarding diagnostic procedures, in patients with suspected pulmonary sequestration or symptoms such as recurrent respiratory tract infections, pneumonia or haemoptysis, chest radiograph represents the first-line exam and can show different types of anomalies, from a lung consolidation, often in the left retrocardiac area, to air–fluid levels caused by bronchial communication, recurrent pneumonia or focal bronchiectasis.^9^ Multidetector CT angiography is the only imaging technique that enables us to clearly identify pulmonary sequestration, to study arterial and venous anatomy depicting vascular anomalies and other possibly related pulmonary and thoracic abnormalities.^2^ Treatment of choice of pulmonary sequestration and its possible related anomalies is selected with a view to prevent the risk of complications, such as massive haemoptysis owing to pulmonary hypertension, congestive heart failure from the left-to-left shunt or severe recurrent infections; surgery is often involved and embolization can be employed before or to substitute surgery.^10^ In the literature, there are only two similar cases where pulmonary sequestration has affected one lung and systemic arterious feeding involved the other normal lung, and they have been described in adult patients.^10^ Ours represents the first paediatric case of intralobar pulmonary sequestration with aspects mimicking the typical pattern of bronchial atresia, associated with a systemic artery supplying the normal contralateral lung.

## Learning points

1. CT scan is essential to correctly identify and classify pulmonary sequestration in the presence of a clinical, prenatal ultrasound or radiographical suspicion.
2. CT angiography is the only non-invasive imaging technique for studying both arterial and venous anatomy in order to identify vascular anomalies that can be associated with pulmonary sequestration.
3. Systemic vascularization of a normal lung is frequently asymptomatic, but sometimes can be responsible for severe manifestations such as haemoptysis or congestive heart failure, and is rarely associated with contralateral pulmonary anomalies; CT angiography is useful in identifying this systemic aberrant vessel.
